# MERS-CoV-3D: integrating whole-genome variation, proteome mapping, and structural annotation of MERS-CoV mutations

**DOI:** 10.1093/database/baag065

**Published:** 2026-10-23

**Authors:** Ali F Alsulami

**Affiliations:** Department of Biochemistry, Faculty of Science, King Abdulaziz University, Jeddah 21589, Saudi Arabia; The Centre for Artificial Intelligence Research & Innovation in Precision Medicines (CAIPM), King Abdulaziz University, Jeddah 21589, Saudi Arabia

## Abstract

Middle East respiratory syndrome coronavirus (MERS-CoV) remains a high-consequence zoonotic pathogen with recurrent outbreak potential and substantially higher reported case fatality than many other human coronaviruses. Although increasing numbers of MERS-CoV genomes and protein structures are available, these data remain distributed across independent sequence, proteome, and structural repositories, limiting systematic interpretation of viral mutations in their functional and three-dimensional contexts. Here, we present an integrated and automated MERS-CoV structural mutation database that connects whole-genome variation, mature viral protein coordinates, experimentally available structures, and residue-level structural annotations. The genome-wide pipeline curates complete or near-complete good-quality MERS-CoV genomes, performs whole-genome alignment, phylogenetic clade and lineage assignment, and identifies amino-acid mutations relative to the reference genome. Replicase mutations are further converted from ORF1a/ORF1ab polyprotein coordinates into mature non-structural protein positions. In parallel, the proteome-wide pipeline automatically retrieves MERS-CoV-related structural records, extracts coordinate-derived chain sequences, performs strict chain-level mapping to the reference proteome, and generates structure-specific mutation mappings. The resulting database contains 1715 unique protein-level mutations, 35 995 cumulative genome-level mutation observations, 456 mapped structure-chain records, 213 unique structures, and 13 542 residue-level structural annotations. The web interface (https://merscov3d.org/version 1.0) lets users browse mutation frequencies, clade distributions, mapped structures, and structural annotations, and inspect mutation positions interactively in a three-dimensional molecular context. Therefore, integrating automated genome surveillance with proteome-scale structural mapping, this resource provides a platform for prioritizing biologically relevant mutations, supporting public health and national surveillance interpretation of emerging MERS-CoV variants, identifying structurally important viral regions, and assisting antiviral-development organizations in drug-target prioritization and resistance-monitoring studies.

## Introduction

Middle East respiratory syndrome coronavirus (MERS-CoV) is a highly pathogenic zoonotic betacoronavirus that was first identified in humans in 2012 and remains an important threat to global public health [1]. Although MERS-CoV has not spread globally at the scale observed for SARS-CoV-2 [2], it continues to cause sporadic human infections, most frequently linked to the Arabian Peninsula, with dromedary camels acting as an important animal reservoir [3]. Human infection can lead to severe lower respiratory disease, pneumonia, acute respiratory distress, multi-organ involvement, and death, particularly in older individuals and patients with underlying comorbidities [4]. Importantly, MERS-CoV also has the capacity to cause healthcare-associated outbreaks, where close contact and insufficient infection-control measures can amplify transmission [5]. Therefore, despite its relatively limited human-to-human transmissibility compared with SARS-CoV-2, MERS-CoV remains a priority pathogen for surveillance, molecular epidemiology, and antiviral research [6].

MERS-CoV is an enveloped, positive-sense, single-stranded RNA virus with a genome of ∼30 kb [7]. Like other coronaviruses, the 5′ region of the genome encodes the large replicase polyproteins open reading frame 1a (ORF1a) and open reading frame 1ab (ORF1ab), which are proteolytically processed into non-structural proteins that form the viral replication–transcription complex [8]. The 3′ region encodes the main structural proteins: spike, envelope, membrane, and nucleocapsid. The spike glycoprotein mediates attachment and entry into host cells through interaction with the cellular receptor dipeptidyl peptidase 4. In addition to its structural and replicase proteins, MERS-CoV encodes accessory proteins, including ORF3, ORF4a, ORF4b, ORF5, and ORF8b [9], which have been implicated in modulation of host innate immune responses and viral pathogenesis [10]. Together, these genomic and proteomic components define the viral life cycle and provide multiple potential targets for surveillance, functional interpretation, and therapeutic intervention [11].

The high mortality associated with MERS-CoV distinguishes it from many other human coronaviruses [12]. Approximately one-third of laboratory-confirmed MERS cases reported to the World Health Organization have resulted in death, although this case-fatality estimate may be affected by under-detection of mild or asymptomatic infections [13]. On the other hand, SARS-CoV-2 caused a far larger global pandemic because of its substantially greater transmissibility, but the reported fatality proportion of COVID-19 has generally been much lower than that reported for MERS-CoV [14, 15]. Thus, MERS-CoV represents a different type of coronavirus threat: one characterized by lower sustained human transmission but markedly higher clinical severity among detected cases [16]. This combination of zoonotic persistence, severe disease outcome, and outbreak potential highlights the need for continued genomic and structural monitoring [17].

Genomic surveillance has transformed the understanding of coronavirus evolution by enabling rapid identification of lineages, mutations, and emerging variants [18]. However, mutation lists alone are often insufficient to interpret biological relevance. Amino-acid substitutions may have very different consequences depending on whether they occur in functional domains, receptor-binding regions, oligomeric interfaces, enzymatic active sites, immune-modulatory proteins, or structurally constrained regions [19, 20]. For MERS-CoV, many genome sequences are available [21, 22], and multiple viral proteins have experimentally solved structures or high-confidence structural information [23–25]. Nevertheless, these data are often distributed across separate genome repositories, protein resources, and structural databases, limiting the ability to connect viral sequence variation directly with protein structure and functional annotation [26–28].

Several coronavirus resources have been developed to support sequence analysis, protein modelling, mutation interpretation, and drug-discovery research [29–31]. During the SARS-CoV-2 pandemic, integrated structural databases provided useful platforms for linking the viral proteome, experimentally solved structures, computational models, binding sites, host interactions, and mutations. A similar integrative approach is valuable for MERS-CoV, particularly because the virus remains endemic in animal reservoirs and continues to generate sporadic human cases. However, compared with SARS-CoV-2, MERS-CoV lacks a comprehensive resource that systematically combines curated whole-genome mutation data, mature protein-level mutation coordinates, experimentally resolved structures, and residue-level structural annotation in an interactive database.

Here, we present a MERS-CoV structural mutation database that integrates genome-wide mutation analysis with proteome-level structural mapping and web-based visualization. Complete or near-complete, good-quality MERS-CoV whole genomes were curated, aligned, and classified into clades and lineages, and amino-acid mutations were called relative to the reference genome. ORF1a and ORF1ab mutations were further mapped onto mature non-structural proteins to provide biologically interpretable protein-level coordinates. In parallel, MERS-CoV proteins were mapped to experimentally available PDB structures at the chain level, and mutation files were generated for structural annotation. The resulting database enables users to browse mutation frequencies, clade and host distributions, mapped structures, and residue-level annotations through an interactive web interface. Therefore, linking MERS-CoV genomic diversity to protein structure, this resource provides a platform for molecular surveillance, hypothesis generation, functional interpretation of mutations, and prioritization of viral proteins or residues for future experimental and therapeutic studies.

## Materials and methods

### Genome-wide pipeline: whole-genome curation, phylogenetic classification, and mutation analysis

A genome-wide bioinformatics pipeline (Fig. 1A) was developed to curate complete or near-complete MERS-CoV whole-genome sequences and identify protein-level mutations relative to the reference genome [1]. Genome metadata were obtained from BV-BRC [32] and standardized to harmonize accession identifiers, genome names, host information, geographic origin, collection year, genome status, genome quality, and sequence length. A total of 771 MERS-CoV genome records were initially retrieved from BV-BRC on [01/07/2026] (supplementary 1). Following quality-control filtering, 199 records were excluded because they represented incomplete or low-quality genomes, short sequences, records without valid GenBank or RefSeq accessions, duplicate accessions, or outgroup-like MERS-related coronavirus sequences. The final curated dataset contained 572 complete or near-complete MERS-CoV genomes (Supplementary 2), which were used for downstream alignment, phylogenetic classification, and mutation calling. The full list of accession numbers used in database version 1.0 is provided as Supplementary Table S1 to support traceability and reproducibility.

**Figure 1:**
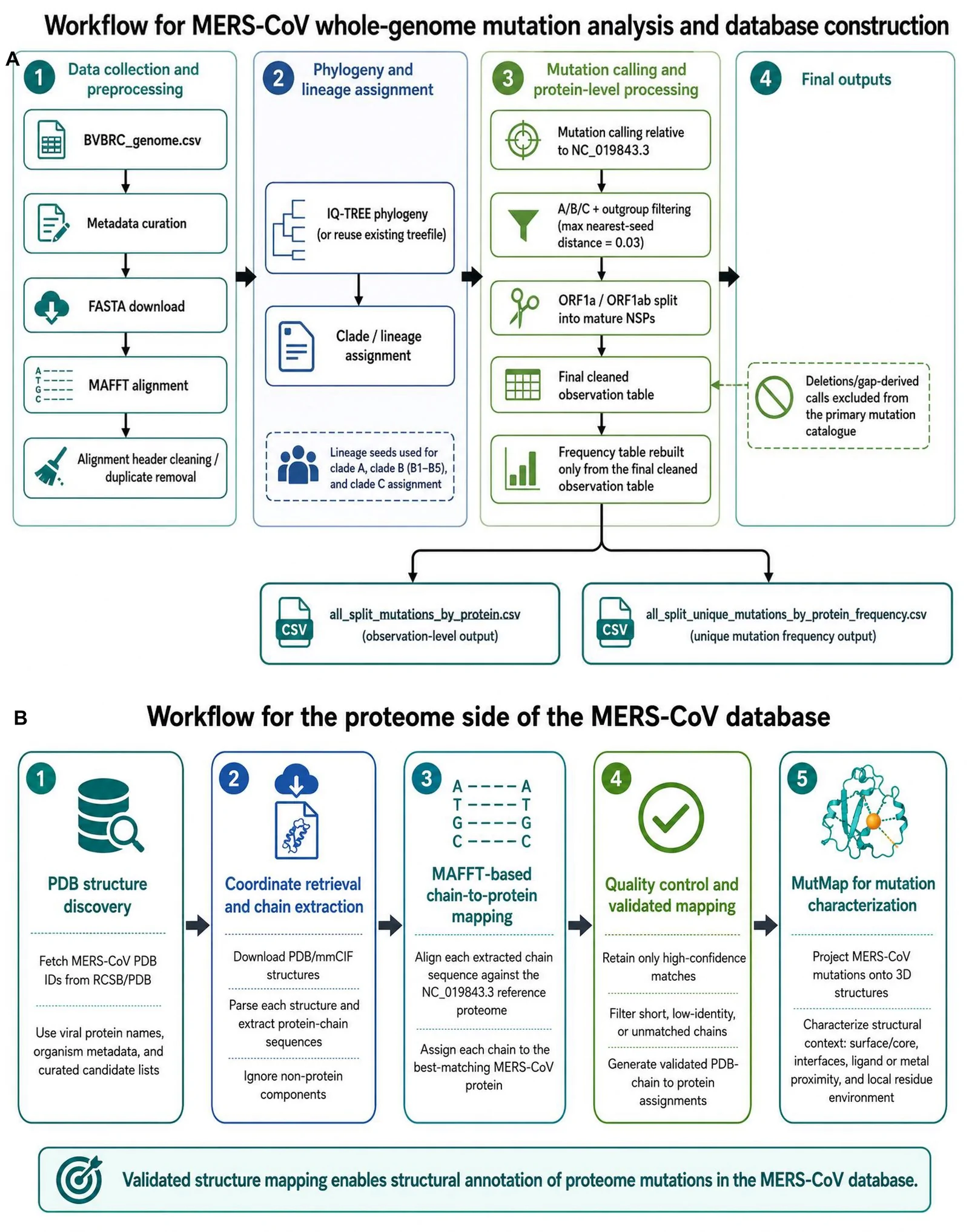
Automated genome-wide and proteome-wide workflow for construction of the MERS-CoV structural mutation database. (A) Illustrates the automated whole-genome mutation analysis workflow. BV-BRC genome metadata were first curated, followed by retrieval of MERS-CoV FASTA sequences, MAFFT-based whole-genome alignment, alignment header cleaning, and duplicate removal. A maximum-likelihood phylogeny was generated using IQ-TREE, and genomes were assigned to MERS-CoV clades and lineages using curated lineage seed sequences. Mutations were then called relative to the reference genome NC_019843.3, followed by filtering to retain clades A, B, and C using a maximum nearest-seed distance threshold of 0.03. ORF1a and ORF1ab mutations were converted into mature non-structural protein coordinates, and the final cleaned observation-level mutation table was used to rebuild the unique mutation-frequency dataset. Gap-derived deletion calls were excluded from the primary mutation catalogue. (B) Illustrates the automated proteome-wide workflow. MERS-CoV-related structures were identified from structural databases, coordinate files were retrieved, protein-chain sequences were extracted, and each chain was mapped to the reference proteome using MAFFT-based chain-to-protein alignment. Only high-confidence mappings were retained after quality control, and validated structures were used for MutMap-based residue-level structural characterization of MERS-CoV mutations. Together, the two automated workflows connect curated genome variation with mature protein coordinates, validated structural mapping, and residue-level annotation for integration into the MERS-CoV-3D database.

The NCBI RefSeq complete genome NC_019843.3, corresponding to MERS-CoV isolate HCoV-EMC/2012, was selected as the reference sequence because it provides a curated and consistently annotated coordinate system for mutation calling and coding-region extraction and is widely used as a standard reference genome in MERS-CoV genomic studies. The selected genomes and the reference sequence were retrieved from NCBI [33], and the reference genome annotation was used to define coding regions. All retained genome sequences were aligned with the reference genome using MAFFT v7.52 [34]. Alignment headers and accession identifiers were normalized, and duplicate sequence records were removed prior to phylogenetic analysis.

A maximum-likelihood phylogeny [35] was inferred from the cleaned whole-genome alignment using IQ-TREE v2.4. Nucleotide substitution model selection was performed using ModelFinder v2.0, and branch support was estimated using ultrafast bootstrap and SH-aLRT procedures. MERS-CoV clade and lineage assignments were then performed using a curated set of lineage seed genomes. Each genome was assigned to the closest labelled seed sequence based on tree distance. To reduce the inclusion of distant MERS-related coronaviruses or outgroup sequences, only genomes assigned to MERS-CoV clades A, B, or C were retained. Additional filtering was applied to remove records whose host, strain name, or genome description indicated bat-associated or MERS-related outgroup viruses. A maximum nearest-seed phylogenetic distance threshold of 0.03 was used to exclude genomes that were too distant from the curated MERS-CoV lineage seeds.

Mutations were called relative to the NC_019843.3 reference genome. For each retained genome, aligned coding regions were reconstructed and compared with the corresponding reference coding sequence. Nucleotide substitutions were reported only when the query base differed from the reference and represented a valid, unambiguous nucleotide. Ambiguous bases and alignment gaps were excluded from nucleotide mutation calling. Amino-acid changes were identified by codon-level comparison and translation of the aligned coding sequence. Codons containing ambiguous nucleotides or alignment gaps were excluded from confident amino-acid mutation calling. This decision was made because alignment gaps in whole-genome datasets may reflect incomplete sequence, low coverage, or assembly uncertainty rather than true biological deletion events. Therefore, the final mutation dataset focused on confident amino-acid substitutions and stop-codon events.

Because the MERS-CoV replicase region is encoded by ORF1a and ORF1ab polyproteins, polyprotein-level mutations were further mapped onto mature non-structural proteins. Reference mature protein sequences were used to convert ORF1a and ORF1ab coordinates into local non-structural protein positions. This generated mature protein-level mutation labels for nsp1–nsp16 and avoided reliance on polyprotein coordinates alone. Duplicate mutation representations arising from the ORF1a/ORF1ab overlap were removed at the genome–protein–mutation level.

The final genome-wide dataset was summarized at both observation and unique-mutation levels. Observation-level records represented individual genomes carrying specific amino-acid mutations, whereas unique-mutation summaries collapsed identical protein-level mutations across genomes. For each unique mutation, the pipeline calculated the number of genomes carrying the mutation and summarized its clade, lineage, host, country, and collection-year distributions. These processed mutation data formed the genomic layer of the MERS-CoV structural mutation resource.

### Proteome-wide pipeline: structural mapping of MERS-CoV proteins

A proteome-wide structural pipeline (Fig. 1B) was developed to identify experimentally resolved MERS-CoV protein structures and connect them to the curated mutation dataset. The MERS-CoV reference proteome was used as the authoritative protein set for all structure-to-protein assignments. Candidate structures were retrieved from the RCSB Protein Data Bank using MERS-CoV-related search terms, including ‘Middle East respiratory syndrome coronavirus’, ‘MERS-CoV’, ‘MERS coronavirus’, and ‘HCoV-EMC’. Metadata associated with each candidate entry, including experimental method, resolution, polymer description, and chain information, were collected to support downstream curation.

Because PDB-level annotations alone can be insufficient for assigning individual chains to viral proteins, final mapping was performed at the coordinate-chain level. Structural coordinate files were downloaded with preference for biological-assembly files when available, followed by deposited coordinate files. Protein chains were extracted from the first model of each structure, and modified residues were converted to their closest standard amino-acid representation where possible. Very short chains were excluded to avoid assigning short peptides or fragments as viral proteins.

Each extracted structure-chain sequence was aligned against the complete MERS-CoV reference proteome using MAFFT v7.52. The best-matching reference protein was identified for each chain, and chains were accepted only when they met a high sequence-identity threshold of at least 98%. This stringent criterion was used to ensure that each structural chain was assigned to the correct viral protein and to prevent misclassification based solely on PDB title, entity description, or deposited metadata. PDB entries containing more than one MERS-CoV protein chain, or more than one viral protein within the same structural entry, were handled at the chain level. This allowed different chains from the same PDB entry to be assigned independently to their corresponding MERS-CoV proteins when appropriate.

For each accepted structural chain, reference protein positions were mapped to residue numbers in the coordinate file. Mutation positions from the genome-wide pipeline were then projected onto the corresponding structure only when the reference residue was present and matched the expected amino acid. Mutations were excluded from structure-specific mapping when the position was unresolved, when the coordinate residue did not match the reference residue, when the structure already contained the mutant amino acid, or when the mutation represented a stop or unknown residue. This residue-level validation ensured that only structurally resolved and biologically interpretable mutations were linked to three-dimensional coordinates.

The proteome-wide pipeline produced a curated structure–protein mapping layer, protein-specific structural datasets, and residue-level mutation mappings using the Mut-Map pipeline v2.0 [36]. These outputs connected MERS-CoV sequence variation with experimentally resolved structural information and enabled downstream structural annotation within the web resource.

### Database development and web implementation

The MERS-CoV structural mutation resource was implemented as a relational database with a Flask-based web interface. The database was designed to integrate three complementary data layers: curated genome-derived mutation frequencies, validated protein–structure mappings, and residue-level structural annotations. Protein names, protein identifiers, structure identifiers, and chain labels were normalized during data loading to ensure consistent linking between mutation records, structural records, and annotation records.

The mutation layer stores mature protein-level amino-acid substitutions and stop-codon events derived from the genome-wide pipeline. For each mutation, the database records the protein name, local amino-acid position, reference residue, alternate residue, mutation type, number of genomes carrying the mutation, and associated epidemiological distributions, including clade, lineage, host, country, and collection-year information. This allows users to examine not only whether a mutation is present, but also how frequently it occurs and how it is distributed across viral clades, hosts, and geographic regions.

The structural layer stores curated links between MERS-CoV proteins and experimentally available structures. Each structure is associated with its mapped viral protein, chain information, experimental method, and resolution when available. Structure identifiers were normalized to allow consistent integration of coordinate files, structural metadata, and residue-level annotation. This layer enables users to identify which viral proteins have structural coverage and which structures can be used to interpret mutation positions.

The annotation layer stores residue-level structural information for mapped mutations. Each annotated residue is linked to its corresponding protein, structure, chain, and residue number, together with mutation frequency and structural annotation class. This design allows mutation records to be queried in the context of protein structure and enables structure-specific browsing of mapped variation.

To support efficient querying, indexes were created on frequently searched fields, including protein identifiers, amino-acid positions, mutation labels, mutation types, structure identifiers, chain identifiers, and annotation classes. Precomputed structure-level summaries were generated to accelerate loading of protein-specific structure pages and annotation summaries. These optimizations allow the web interface to display mutation and structure data interactively without repeatedly performing expensive joins during user requests.

The web application was developed using Flask and provides protein-level, mutation-level, and structure-level access to the database. The home page summarizes the available MERS-CoV proteins, mutations, structures, and mapped annotations. Protein-specific pages provide interactive tables for browsing mutation frequencies and associated structures. Server-side table processing was implemented to support searching, sorting and pagination of large datasets. Structure-specific pages display mapped residue annotations and summary charts for structural annotation classes.

Three-dimensional molecular visualization was integrated using Mol* [37]. Structure files are served through dedicated web routes and displayed in an embedded viewer. Residue-level annotations are linked to the structural viewer, allowing users to inspect mapped mutation positions in their three-dimensional context. The interface was designed to support rapid exploration of MERS-CoV genomic variation, protein-level mutation frequencies, clade and host distributions, experimentally resolved structures and residue-level structural annotations within a single web resource.

## Results

### Genome and proteome analysis

The curated MERS-CoV mutation dataset contained 1715 unique protein-level amino-acid mutations distributed across 25 viral proteins. These mutations represented 35 995 cumulative genome-level mutation observations, indicating that several mutations were repeatedly observed across multiple genomes. The mutation spectrum was dominated by missense substitutions, which accounted for 1704 mutations, whereas stop-codon events were rare, accounting for only 11 mutations. This pattern indicates that the database mainly captures amino-acid substitutions rather than protein-truncating changes, consistent with the filtering strategy used to retain confident protein-level variants from complete or near-complete genomes.

Mutation burden was unevenly distributed across the MERS-CoV proteome. The largest number of unique mutations was observed in nsp3, with 384 unique amino-acid changes, followed by the spike protein with 282 mutations and nsp2 with 135 mutations. Together, nsp3 and spike accounted for 666 unique mutations, representing ∼38.8% of all unique mutations in the dataset (Fig. 2B). Other proteins with notable mutation counts included the nucleocapsid protein, NS4B, NS3, nsp12, nsp4, nsp14, and nsp15. When mutation burden was weighted by genome-level observations, nsp3 remained the most mutation-rich protein, accounting for 11 511 observations, followed by nsp2, NS3, spike, and nsp4 (Fig. 2A). This indicates that nsp3 is not only diverse at the level of unique mutation sites but also contains mutations that recur frequently across genomes.

**Figure 2:**
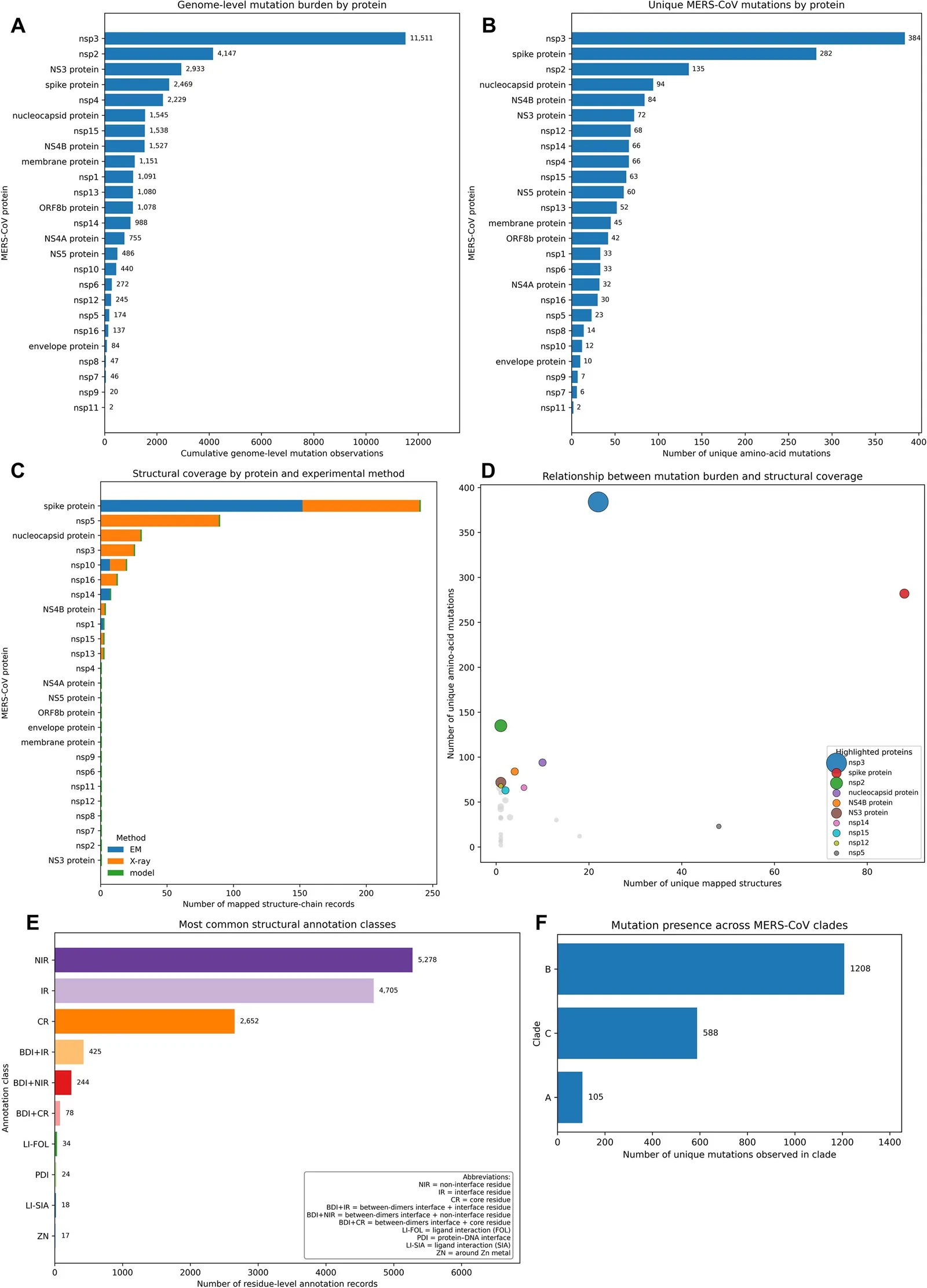
Genome-wide mutation distribution and structural coverage of the MERS-CoV proteome. Integrated genome and proteome analysis of the curated MERS-CoV structural mutation database. (A) Cumulative genome-level mutation observations by viral protein, reflecting the recurrence of mutations across the curated genome dataset. (B) Distribution of unique protein-level amino-acid mutations across MERS-CoV proteins, showing the highest mutation burden in nsp3 and spike protein. (C) Structural coverage of MERS-CoV proteins, shown as mapped structure-chain records grouped by experimental method. (D) Relationship between mutation burden and structural coverage across MERS-CoV proteins. Each point represents one viral protein; highlighted proteins are identified in the in-figure legend, while other proteins are shown in grey. Point size reflects the cumulative number of genome-level mutation observations. (E) Residue-level structural annotation classes assigned to mapped mutation positions. Abbreviations: NIR, non-interface residue; IR, interface residue; CR, core residue; BDI, between-dimers interface; LI-FOL, ligand interaction with FOL; PDI, protein–DNA interface; LI-SIA, ligand interaction with SIA; ZN, residue located around zinc metal. (F) Distribution of unique mutations across MERS-CoV clades A, B, and C. Counts represent mutation presence within each clade and are not mutually exclusive because some mutations occur in more than one clade. Together, these analyses show that MERS-CoV mutation burden is unevenly distributed across the proteome and that structural coverage varies substantially between highly mutated and structurally well-characterized proteins.

Analysis of clade distribution showed that most unique mutations were observed in clade B genomes (Fig. 2E). A total of 1208 unique mutations were associated with clade B, compared with 588 mutations associated with clade C and 105 associated with clade A. Because some mutations were present in more than one clade, these counts represent clade presence rather than mutually exclusive mutation counts. The broader representation of clade B mutations is consistent with the larger contribution of clade B genomes to the curated MERS-CoV dataset and highlights the importance of clade-aware interpretation when evaluating mutation frequency.

The proteome-wide structural analysis identified 456 mapped structure-chain records corresponding to 213 unique PDB or model structures across the MERS-CoV proteome. Structural coverage was highly enriched for the spike protein, which accounted for 241 mapped structure-chain records and 88 unique structures. The next most represented protein was nsp5, with 90 mapped structure-chain records and 48 unique structures, followed by the nucleocapsid protein, nsp3, nsp10, and nsp16. Experimental methods were dominated by X-ray crystallography, which represented 263 structure-chain records, followed by electron microscopy with 168 records (Fig. 2C). Model-derived structures accounted for 25 records and provided structural coverage for proteins with limited or absent experimental structural representation.

Integration of mutation and structure data revealed that structural coverage is not evenly proportional to mutation burden. For example, spike had both high mutation diversity and extensive structural coverage, making it a strong candidate for detailed structure-guided mutation analysis. In contrast, nsp3 showed the highest number of unique mutations but had more limited structural coverage relative to its mutation burden. Conversely, nsp5 had extensive structural representation despite a smaller number of observed mutations (Fig. 2D). This imbalance highlights the value of integrating genome-derived mutation frequencies with proteome-level structural coverage, because proteins with high mutational diversity may not always be the proteins with the richest structural data.

Residue-level structural annotation produced 13 542 mapped annotation records. The most common annotation classes were non-interface residues, interface residues, and core residues, accounting for 5278, 4705, and 2652 records, respectively. Additional annotations included residues located at between-dimer interfaces, protein–DNA interfaces, metal-proximal residues, and ligand-interacting residues (Fig. 2F). These annotations provide a structural context for interpreting MERS-CoV mutations beyond simple protein position, enabling users to prioritize mutations that occur at interfaces, buried regions, ligand-binding environments, or other structurally relevant sites.

Overall, the genome and proteome analyses demonstrate that the database captures both the breadth of MERS-CoV sequence variation and the structural context required for biological interpretation. The resource links mutation frequency, clade distribution, protein identity, structural coverage, and residue-level annotation in a single framework, providing a foundation for exploring mutations with potential effects on viral protein stability, molecular interactions, immune recognition, and antiviral target sites.

### Front-end organization of the MERS-CoV database

The MERS-CoV database front end was organized into three main levels: the main page, the gene/protein page and the structural page. The main page provides an entry point to the resource and summarizes the overall content of the database, including the available MERS-CoV proteins, curated mutations, mapped structures, and structural annotations. Users can browse the viral proteome and select a protein of interest to access its associated mutation and structural information.

The gene/protein page provides a protein-specific view of the database. For each selected MERS-CoV protein, the page displays the curated list of amino-acid mutations with their local protein coordinates, mutation type, genome count, and associated epidemiological distributions, including clade, lineage, host, country, and collection-year information. The same page also presents the available mapped structures for that protein, allowing users to move directly from mutation-level information to structure-based interpretation (Fig. 3A).

**Figure 3:**
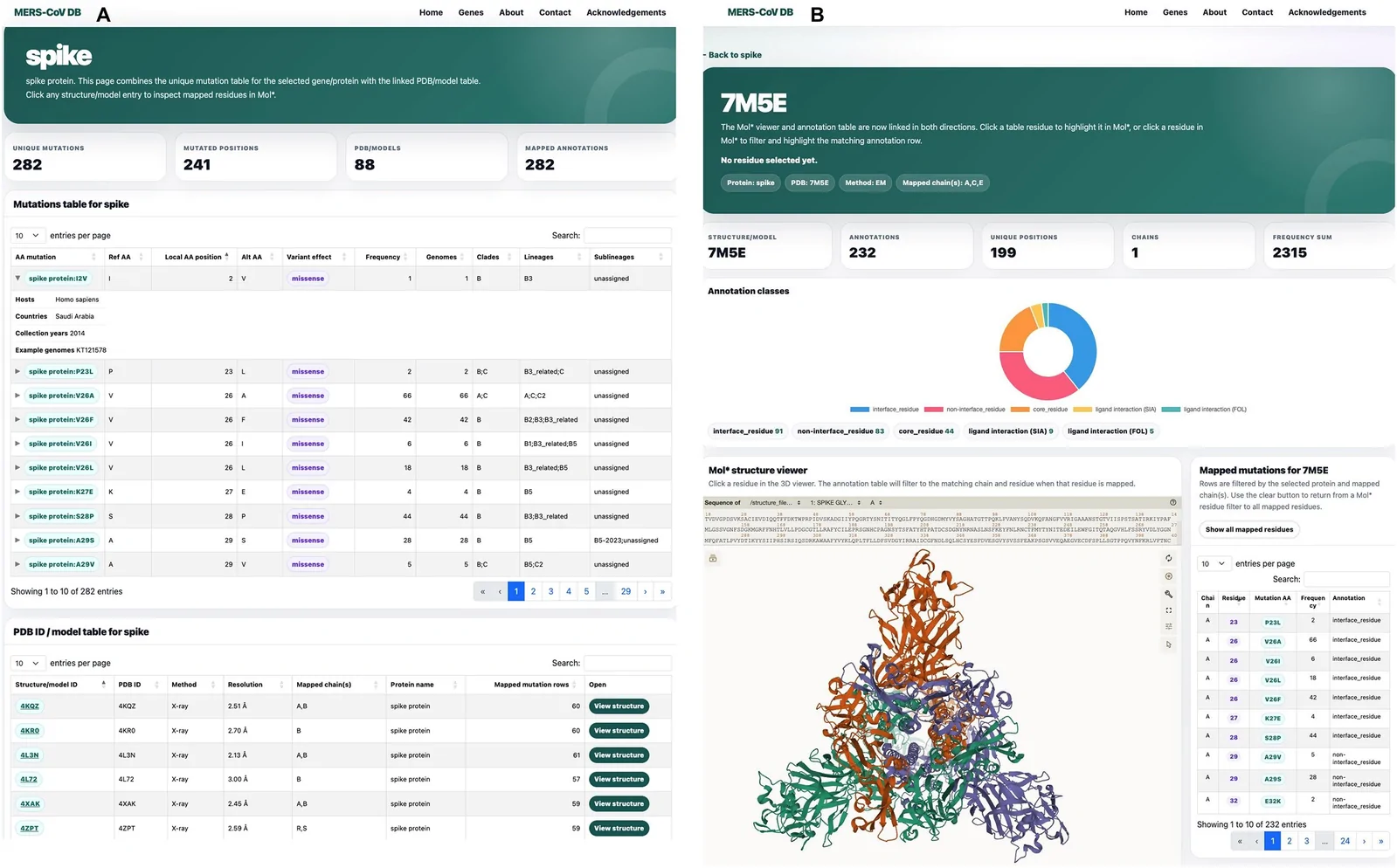
Web interface of the MERS-CoV-3D structural mutation database. (A) The gene/protein page, shown here for spike, summarizes the selected protein and displays mutation statistics, including the number of curated mutations, mapped structures, unique structures, and total mutation observations. The page also provides interactive mutation and structure tables, enabling users to examine local amino-acid positions, mutation types, frequencies, clade/lineage distributions, and linked structural models. (B) The structure page, shown here for PDB entry 7M5E, provides residue-level structural interpretation for a selected structure. The page summarizes mapped annotations, unique positions, chain information, and frequency totals and displays annotation-class distributions. An embedded Mol* viewer allows mapped residues to be inspected in three-dimensional context, while the linked mutation table enables users to identify residue positions, amino-acid changes, mutation frequencies, and structural annotation classes. Together, these pages allow users to move from global proteome-level browsing to protein-specific mutation analysis and structure-level interpretation of MERS-CoV variation.

The structural page provides a residue-level view for each selected structure. This page links mapped mutations and structural annotations to the corresponding three-dimensional coordinate file. Residue annotations are displayed in an interactive table and are connected to an embedded Mol* molecular viewer, enabling users to inspect mutation positions within their structural context. This allows rapid evaluation of whether a mutation occurs in a core region, surface region, protein–protein interface, ligand-binding environment, metal-proximal site, or other annotated structural feature. Together, these three interface levels allow users to move from global proteome-level summaries to protein-specific mutation data and finally to structure-level residue interpretation (Fig. 3B).

## Discussion

MERS-CoV remains an important coronavirus threat because it combines zoonotic persistence, severe clinical outcome, and recurrent outbreak potential [16, 38]. Although it has not generated a pandemic on the scale of SARS-CoV-2, its continued circulation in animal reservoirs and its ability to cause severe human disease justify sustained genomic and structural surveillance. The database presented here addresses this need by linking MERS-CoV sequence variation to mature viral proteins, experimentally resolved structures, and residue-level structural annotations. This integration is important because mutation frequency alone does not provide sufficient biological interpretation. A mutation observed in many genomes may have limited functional impact if it occurs in a flexible or non-conserved surface region, whereas a rarer mutation may be more important if it occurs in an enzyme active site, oligomeric interface, ligand-binding pocket, or structurally constrained core region.

A major value of this resource is that it transforms genome-derived mutations into structure-guided biological hypotheses. Therefore, by mapping mutations onto experimentally available structures, the database allows users to distinguish mutations that are likely to be structurally neutral from those that may affect protein stability, molecular recognition, enzymatic activity, or protein–protein interactions. For example, mutations located in core regions may suggest effects on local folding or protein stability, whereas interface mutations may suggest altered oligomerization, host-factor binding, or viral protein complex formation. Mutations near ligand-binding or metal-proximal regions may be particularly relevant for antiviral target prioritization, because such changes could influence inhibitor binding, catalytic geometry. Therefore, the database is not only a catalogue of mutations but also a platform for generating experimentally testable mechanistic hypotheses.

The resource is also valuable for drug-discovery research. Several MERS-CoV proteins, including viral proteases, polymerase-associated proteins, and spike, are attractive targets for therapeutic intervention [39, 40]. However, antiviral development requires consideration of both structural druggability and natural viral variation. A binding pocket that appears suitable for inhibitor design may be less attractive if it contains highly variable residues, whereas a conserved and structurally constrained pocket may represent a more robust drug target. Therefore, combining mutation frequency with structural annotation, the database can help identify residues and regions that are both structurally important and less tolerant to variation. Conversely, it can highlight variable regions that may require caution during inhibitor optimization or antibody-design studies. This makes the resource useful for prioritizing residues for docking, molecular dynamics simulations, mutagenesis experiments, and resistance-risk assessment.

The database can also support future surveillance of MERS-CoV evolution every two years. As additional genomes become available, newly observed mutations can be incorporated and rapidly interpreted in the context of existing protein structures. This is particularly important for mutations that arise in spike, polymerase-associated proteins, proteases, or accessory proteins involved in host interaction and immune modulation. A future mutation that appears only once in a genome dataset may still deserve attention if it maps to a receptor-binding region, enzymatic pocket, interface, or structurally conserved site. In this way, the database provides a framework for moving beyond simple mutation counting towards biologically informed mutation tracking.

Another important aspect of this work is the mapping of ORF1a and ORF1ab variation onto mature non-structural proteins. Coronavirus replicase mutations are often difficult to interpret when reported only in polyprotein coordinates. Therefore, by converting these mutations into mature nsp-level positions, the database makes the data more accessible to researchers studying individual viral enzymes and replication-complex components. This is particularly useful for structural biology and drug discovery, where functional interpretation is usually performed at the level of mature proteins rather than polyprotein coordinates.

The resource also highlights the uneven relationship between mutation burden and structural coverage across the MERS-CoV proteome. Some proteins, such as spike, have both high mutational diversity and strong structural representation, making them suitable for immediate structure-guided analysis. Other proteins, such as nsp3, carry a high mutation burden but have more limited structural coverage relative to their size and diversity. This mismatch identifies areas where future structural studies would be especially valuable. Conversely, proteins with extensive structural coverage but lower observed mutation burden may represent conserved and potentially attractive therapeutic targets. Thus, the database can guide not only mutation interpretation but also future experimental structural biology priorities.

Despite these strengths, several limitations should be acknowledged. First, the database depends on the quality and completeness of publicly available genome sequences. Under-sampling of certain hosts, countries, or time periods may bias apparent mutation frequencies and clade distributions. Second, structural interpretation is limited by the availability and completeness of experimentally resolved structures. Missing loops, unresolved domains, and incomplete assemblies may prevent some mutations from being mapped to coordinates. Third, structural annotation provides biological context but does not by itself prove functional impact. Mutations highlighted by the database should therefore be followed up using complementary approaches, such as conservation analysis, free-energy prediction, biochemical assays, reverse genetics, docking, or molecular dynamics simulations.

Future development of the database could include automated periodic genome updates, integration of conservation scores, prediction of mutation effects on protein stability and binding, mapping of mutations to functional domains and epitopes, and incorporation of molecular dynamics or docking-derived annotations. Integration with antiviral screening data could further strengthen the drug-discovery utility of the resource by linking natural viral variation to experimentally tested compounds and binding sites. Such extensions would allow the database to evolve from a structural mutation catalogue into a broader platform for MERS-CoV molecular surveillance and therapeutic hypothesis generation.

In summary, this database provides an integrated resource for interpreting MERS-CoV mutations in their mature protein and structural contexts. Therefore, connecting genome surveillance, proteome mapping, and residue-level structural annotation, it enables users to prioritise mutations, identify structurally important viral regions, generate drug-discovery hypotheses, and monitor future viral variation in a biologically informed manner. This framework may also serve as a model for developing similar structural mutation resources for other emerging or re-emerging RNA viruses.

## Conclusion

MERS-CoV-3D is a structural mutation database that connects curated whole-genome variation with mature viral protein coordinates, experimentally available structural data, and residue-level annotation. Therefore, combining genome-wide mutation analysis with proteome-wide structural mapping, the resource allows MERS-CoV mutations to be interpreted beyond sequence position alone and placed within biologically meaningful contexts such as protein cores, interfaces, ligand-binding regions, and other structurally relevant sites.

The database provides a practical platform for exploring MERS-CoV diversity, identifying recurrent or structurally important mutations, comparing mutation burden across viral proteins, and assessing the relationship between genomic variation and structural coverage. This is particularly valuable for proteins involved in viral entry, replication, immune modulation, and antiviral targeting. The resource can support future molecular surveillance, guide experimental prioritization and generate structure-based hypotheses for drug discovery and resistance monitoring.

As additional MERS-CoV genomes and structures become available, the database can be expanded to provide an increasingly comprehensive view of viral evolution and functional variation. More broadly, this work demonstrates the value of integrating genomic surveillance with structural bioinformatics for emerging and re-emerging RNA viruses.

## Supplementary Material

baag065_Supplemental_Files

## References

[1] Zaki AM, van Boheemen S, Bestebroer TM et al. Isolation of a novel coronavirus from a man with pneumonia in Saudi Arabia. N Engl J Med. 2012; 367:1814–20. 10.1056/NEJMoa1211721 23075143

[2] Li J, Lai S, Gao GF et al. The emergence, genomic diversity and global spread of SARS-CoV-2. Nature. 2021; 600:408–18. 10.1038/s41586-021-04188-6 34880490

[3] Aly M, Elrobh M, Alzayer M et al. Occurrence of the Middle East respiratory syndrome coronavirus (MERS-CoV) across the Gulf Corporation Council countries: four years update. PLoS One. 2017; 12:e0183850. 10.1371/journal.pone.0183850 PMC5640221 29028812

[4] Thankamani K, Shubham D, Kandpal G et al. Middle East respiratory syndrome coronavirus (MERS-CoV) internalization does not rely on DPP4 cytoplasmic tail signaling. Npj Viruses. 2024; 2:67. 10.1038/s44298-024-00080-y PMC11721135 40295839

[5] Baharoon S, Memish ZA. MERS-CoV as an emerging respiratory illness: a review of prevention methods. Travel Med Infect Dis. 2019; 32:101520. 10.1016/j.tmaid.2019.101520 PMC7110694 31730910

[6] Tan CCS, Lam SD, Richard D et al. Transmission of SARS-CoV-2 from humans to animals and potential host adaptation. Nat Commun. 2022; 13:2988. 10.1038/s41467-022-30698-6 PMC9142586 35624123

[7] Schroeder S, Mache C, Kleine-Weber H et al. Functional comparison of MERS-coronavirus lineages reveals increased replicative fitness of the recombinant lineage 5. Nat Commun. 2021; 12:5324. 10.1038/s41467-021-25519-1 PMC8423819 34493730

[8] de Wit E, van Doremalen N, Falzarano D et al. SARS and MERS: recent insights into emerging coronaviruses. Nat Rev Micro. 2016; 14:523–34. 10.1038/nrmicro.2016.81 PMC7097822 27344959

[9] Durai P, Batool M, Shah M et al. Middle East respiratory syndrome coronavirus: transmission, virology and therapeutic targeting to aid in outbreak control. Exp Mol Med. 2015; 47:e181–81. 10.1038/emm.2015.76 PMC4558490 26315600

[10] Faure E, Poissy J, Goffard A et al. Distinct immune response in two MERS-CoV-infected patients: can we go from bench to bedside?. PLoS One. 2014; 9:e88716–. 10.1371/journal.pone.0088716 PMC3925152 24551142

[11] Zumla A, Chan JFW, Azhar EI et al. Coronaviruses—drug discovery and therapeutic options. Nat Rev Drug Discovery. 2016; 15:327–47. 10.1038/nrd.2015.37 PMC7097181 26868298

[12] Hui DS, Azhar EI, Kim YJ et al. Middle East respiratory syndrome coronavirus: risk factors and determinants of primary, household, and nosocomial transmission. Lancet Infect Dis. 2018; 18:e217–27. 10.1016/S1473-3099(18)30127-0 PMC7164784 29680581

[13] Azhar EI, Hui DSC, Memish ZA et al. The Middle East respiratory syndrome (MERS). Infect Dis Clin North Am. 2019; 33:891–905. 10.1016/j.idc.2019.08.001 PMC7127753 31668197

[14] Anderson RM, Vegvari C, Hollingsworth TD et al. The SARS-CoV-2 pandemic: remaining uncertainties in our understanding of the epidemiology and transmission dynamics of the virus, and challenges to be overcome. Interface Focus. 2021; 11:20210008. 10.1098/rsfs.2021.0008 PMC8504893 34956588

[15] Asokan S, Isiaka ID, Jacob T et al. Middle East respiratory syndrome coronavirus (MERS-CoV): an underestimated betacoronavirus with pandemic potential. Diagn Microbiol Infect Dis. 2026; 115:117367. 10.1016/j.diagmicrobio.2026.117367 41831387

[16] Al Sulayyim HJ, Khorshid SM, Al Moummar SH. Demographic, clinical, and outcomes of confirmed cases of Middle East respiratory syndrome coronavirus (MERS-CoV) in Najran, Kingdom of Saudi Arabia (KSA); A retrospective record based study. J Infect Public Health. 2020; 13:1342–46. 10.1016/j.jiph.2020.04.007 PMC7180355 32354534

[17] Subissi L, Otieno JR, Shah AAM et al. Human MERS-CoV cases are falling but pose an ongoing pandemic threat. Nat Health. 2026; 1:675–678. 10.1038/s44360-026-00098-x

[18] Al-Tawfiq JA, Assiri AM Persistent signals from MERS-CoV in 2025: surveillance gaps, clinical heterogeneity, and preparedness priorities. New Microbes New Infect. 2026; 70:101704. 10.1016/j.nmni.2026.101704 PMC12860262 41626525

[19] Krismer L, Schöppe H, Rauch S et al. Study of key residues in MERS-CoV and SARS-CoV-2 main proteases for resistance against clinically applied inhibitors nirmatrelvir and ensitrelvir. Npj Viruses. 2024; 2:23. 10.1038/s44298-024-00028-2 PMC11196219 38933182

[20] Hannah K-W, Elzayat MT, Wang L et al. Mutations in the spike protein of Middle East respiratory syndrome coronavirus transmitted in Korea increase resistance to antibody-mediated neutralization. J Virol. 2019; 93:e01381–18. 10.1128/jvi.01381-18 PMC6321919 30404801

[21] Farrag MA, Amer HM, Bhat R et al. Sequence and phylogentic analysis of MERS-CoV in Saudi Arabia, 2012–2019. Virol J. 2021; 18:90. 10.1186/s12985-021-01563-7 PMC8085657 33931099

[22] Kandeil A, Shehata MM, El Shesheny R et al. Complete genome sequence of Middle East respiratory syndrome coronavirus isolated from a dromedary camel in Egypt. Genome Announc. 2016; 4:e00309–16. 10.1128/genomea.00309-16 PMC4850855 27125484

[23] Yuan Y, Cao D, Zhang Y et al. Cryo-EM structures of MERS-CoV and SARS-CoV spike glycoproteins reveal the dynamic receptor binding domains. Nat Commun. 2017; 8:15092. 10.1038/ncomms15092 PMC5394239 28393837

[24] Pallesen J, Wang N, Corbett KS et al. Immunogenicity and structures of a rationally designed prefusion MERS-CoV spike antigen. Proc Natl Acad Sci. 2017; 114:E7348–57. 10.1073/pnas.1707304114 PMC5584442 28807998

[25] Needle D, Lountos GT, Waugh DS Structures of the Middle East respiratory syndrome coronavirus 3C-like protease reveal insights into substrate specificity. Acta Crystallogr D Biol Crystallogr. 2015; 71:1102–11. 10.1107/S1399004715003521 PMC4427198 25945576

[26] Burley SK, Bhatt R, Bhikadiya C et al. Updated resources for exploring experimentally-determined PDB structures and Computed Structure Models at the RCSB Protein Data Bank. Nucleic Acids Res. 2025; 53:D564–74. 10.1093/nar/gkae1091 PMC11701563 39607707

[27] Benson DA, Cavanaugh M, Clark K et al. GenBank. Nucleic Acids Res. 2013; 41:D36–42. 10.1093/nar/gks1195 PMC3531190 23193287

[28] Consortium, T. U. UniProt: the universal protein knowledgebase in 2021. Nucleic Acids Res. 2021; 49:D480–89. 10.1093/nar/gkaa1100 PMC7778908 33237286

[29] Alsulami AF, Thomas SE, Jamasb AR et al. SARS-CoV-2 3D database: understanding the coronavirus proteome and evaluating possible drug targets. Briefings Bioinf. 2021; 22:769–80. 10.1093/bib/bbaa404 PMC7929435 33416848

[30] Portelli S, Olshansky M, Rodrigues CHM et al. Exploring the structural distribution of genetic variation in SARS-CoV-2 with the COVID-3D online resource. Nat Genet. 2020; 52:999–1001. 10.1038/s41588-020-0693-3 32908256

[31] Wlodawer A, Dauter Z, Shabalin IG et al. Ligand-centered assessment of SARS-CoV-2 drug target models in the Protein Data Bank. FEBS J. 2020; 287:3703–18. 10.1111/febs.15366 PMC7276724 32418327

[32] Shukla M, Wattam AR, Aleman A et al. BV-BRC: a unified bacterial and viral bioinformatics resource with expanded functionality and AI integration. Nucleic Acids Res. 2026; 54:D715–23. 10.1093/nar/gkaf1254 PMC12807693 41251138

[33] O’Leary NA, Cox E, Holmes JB et al. Exploring and retrieving sequence and metadata for species across the tree of life with NCBI Datasets. Sci Data. 2024; 11:732. 10.1038/s41597-024-03571-y PMC11226681 38969627

[34] Katoh K, Standley DM MAFFT multiple sequence alignment software version 7: improvements in performance and usability. Mol Biol Evol. 2013; 30:772–80. 10.1093/molbev/mst010 PMC3603318 23329690

[35] De Maio N, Kalaghatgi P, Turakhia Y et al. Maximum likelihood pandemic-scale phylogenetics. Nat Genet. 2023; 55:746–52. 10.1038/s41588-023-01368-0 PMC10181937 37038003

[36] Alsulami AF Mut-Map: comprehensive computational pipeline for structural mapping and analysis of cancer-associated mutations. Briefings Bioinf. 2024; 25:bbae514. 10.1093/bib/bbae514 PMC11483132 39413799

[37] Sehnal D, Bittrich S, Deshpande M et al. Mol* Viewer: modern web app for 3D visualization and analysis of large biomolecular structures. Nucleic Acids Res. 2021; 49:W431–37. 10.1093/nar/gkab314 PMC8262734 33956157

[38] Bernard-Stoecklin S, Nikolay B, Assiri A et al. Comparative analysis of eleven healthcare-associated outbreaks of Middle East respiratory syndrome coronavirus (Mers-Cov) from 2015 to 2017. Sci Rep. 2019; 9:7385. 10.1038/s41598-019-43586-9 PMC6517387 31089148

[39] Al-Tawfiq JA, Memish ZA. What are our pharmacotherapeutic options for MERS-CoV?. Expert Rev Clin Pharmacol. 2014; 7:235–38. 10.1586/17512433.2014.890515 24580083

[40] Omrani AS, Memish ZA Therapeutic options for Middle East respiratory syndrome coronavirus (MERS-CoV) infection: how close are we?. Curr Treat Options Infect Dis. 2015; 7:202–16. 10.1007/s40506-015-0048-2 PMC7100761 32226324

